# In vitro inhibition of porcine reproductive and respiratory syndrome virus replication by short antisense oligonucleotides with locked nucleic acid modification

**DOI:** 10.1186/s12917-018-1432-1

**Published:** 2018-03-26

**Authors:** Lingyun Zhu, Junlong Bi, Longlong Zheng, Qian Zhao, Xianghua Shu, Gang Guo, Jia Liu, Guishu Yang, Jianping Liu, Gefen Yin

**Affiliations:** 1 Department of Veterinary Medicine, College of Animal Science and Technology, Yunnan Agricultural University, Yunnan province, Kunming, 650201 China; 2 Haikou Experimental Station/Hainan Key Laboratory of Banana Genetic Improvement, Chinese Academy of Tropical Agricultural Sciences, Haikou, 570102 Hainan People’s Republic of China; 3 Department of Medical Biochemistry and Biophysics, Karolinska Institute, -17177 Stockholm, SE Sweden; 4 Present address: Center for Animal Disease Control and Prevention, City, 675000, Yunnan province, Chuxiong, China; 5 Present address: College of Animal Science and Technology, Shanxi Agricultural University, Shanxi province, Taigu, 030801 China

**Keywords:** Porcine reproductive and respiratory syndrome virus (PRRSV), Locked nucleic acids (LNAs), Virus replication, Antisense oligonucleotides, Marc-145, Pulmonary alveolar macrophages (PAMs)

## Abstract

**Background:**

Porcine reproductive and respiratory syndrome virus (PRRSV) causes porcine reproductive and respiratory syndrome (PRRS), which is currently insufficiently controlled. From a previous small-scale screen we identified an effective DNA-based short antisense oligonucleotide (AS-ON) targeting viral NSP9, which could inhibit PRRSV replication in both Marc-145 cells and pulmonary alveolar macrophages (PAMs). The objective of this study was to explore the strategy of incorporating locked nucleic acids (LNAs) to achieve better inhibition of PRRSV replication in vitro.

**Methods:**

The effective DNA-based AS-ON (YN8) was modified with LNAs at both ends as gap-mer (LNA-YN8-A) or as mix-mer (LNA-YN8-B). Marc-145 cells or PAMs were infected with PRRSV and subsequently transfected.

**Results:**

Compared with the DNA-based YN8 control, the two AS-ONs modified with LNAs were found to be significantly more effective in decreasing the cytopathic effect (CPE) induced by PRRSV and thus in maintaining cell viability. LNA modifications conferred longer lifetimes to the AS-ON in the cell culture model. Viral ORF7 levels were more significantly reduced at both RNA and protein levels as shown by quantitative PCR, western blot and indirect immunofluorescence staining. Moreover, transfection with LNA modified AS-ON reduced the PRRSV titer by 10-fold compared with the YN8 control.

**Conclusion:**

Taken together, incorporation of LNA into AS-ON technology holds higher therapeutic promise for PRRS control.

## Background

Porcine reproductive and respiratory syndrome (PRRS) is characterized by respiratory disorders in piglets and reproductive failure in sows [1]. The disease is one of the most economically significant problems in the swine industry. The responsible virus, porcine reproductive and respiratory syndrome virus (PRRSV) is a member of the family *Arteriviridae*, genus *Arterivirus* [2]. PRRSV is an enveloped, single-stranded positive-sense RNA virus. PRRSV’s genome is about 15 kb long, consisting of nine open reading frames (ORFs) [3]. Among all the encoded viral proteins, NSP9 is a putative RNA-dependent RNA polymerase and plays central roles in viral replication [4]. In our previous small screen with nine candidate sequences [5], we identified YN8 as one of the most effective DNA-based short antisense oligonucleotides (AS-ONs) targeting NSP9, which could significantly inhibit PRRSV replication in both Marc-145 cells and pulmonary alveolar macrophages (PAMs).

Due to the disadvantages of DNA-based AS-ON, e.g. relatively low biostability, quick degradation by nucleases and low hybridization affinity with target sequences, the applications of antisense technologies in research and therapeutics are limited. The synthesis of Locked Nucleic Acid (LNA) [6] overcame these limitations as LNA modified nucleotides confer low cytotoxicity, high thermostability, resistance to nucleases and stable hybridization abilities with target sequences [7]. Enhanced nucleic acid recognition by LNA-containing oligonucleotides made them desirable for many applications in molecular biology, including genotyping [8] or single nucleotide polymorphism (SNP) analysis [9], hybridization [10, 11], decoy and fluorescence polarization [12], expression profiling or microarray [13], allele-specific PCR [14], fluorescent in situ hybridization (FISH) analysis [15], alteration of intron splicing and LNAzymes [16], 5′-nuclease assay [17], real-time PCR [18], siRNA [19], microRNA [20] and antisense [21].

In 2006, highly pathogenic PRRSV strains of the North American type were identified in more than 10 provinces in China, where they caused approximately four million fatal cases in 2006 [22]. At the beginning of 2007, the disease re-emerged and infected 310,000 pigs, of which more than 81,000 died in 26 provinces [23].The outbreak of PRRS in China has resulted in considerable economic losses and a rise in the price of pork. Therefore, it is urgent to develop more effective strategies to prevent and control PRRSV infection in the swine industry. In order to develop improved methods to manage PRRS, we selected the best antisense sequence YN8 from our previous small-scale screening [5] for LNA modifications and applied the two modified sequences to in vitro studies (lifetime of the antisense oligonucleotides, cytotoxicity, cytopathic effect observation, qPCR, virus titer assessment, western blot and indirect immunofluorescence) to evaluate the inhibitory effects on PRRSV replication in Marc-145 cells and in PAMs between the DNA- and LNA-based AS-ONs. Our data showed that incorporation of LNA into AS-ON technology holds higher therapeutic promise for PRRS control.

## Methods

### Ethics statement

In this study the pigs did not undergo any manipulations prior to standard industrial slaughter. Therefore, no specific ethical approval was required. All animal experiments were performed with the approval of the Animal Care Committee of Yunnan Agricultural University, China.

### Virus and cells

From the lungs of an infected pig in Yunnan province (China) during a severe PRRSV outbreak in 2008, our research group isolated a highly pathogenic PRRSV field strain YN-1 (GenBank accession number: KJ747052), which belongs to the North American genotype. Both pulmonary alveolar macrophages (PAMs) and Marc-145 cells were applied in this study, as PRRSV can replicate in these two culture systems. The Marc-145 cells and PAMs were acquired and cultured as we previously described [5].

### Locked nucleic acid modification in antisense oligonucleotide sequences

According to the statistic conclusion [24], we designed nine candidate AS-ONs with a length of 20 nt using RNA Structure 5.6 [25, 26] and YN8 was identified as the best antisense sequence inhibiting the replication of PRRSV in vitro [5]. In the present study, three types of antisense oligonucleotides based on YN-8 were investigated: (i) unmodified AS-ON (DNA, YN8, served as a control); (ii) LNA/DNA/LNA gap-mer with four LNAs at both ends (LNA-YN8-A) and (iii) LNA/DNA mix-mer (LNA-YN8-B). The three AS-ONs are listed in Table 1 and were synthesized by Shengong (Shanghai, China). The nucleotides in bold in the antisense oligonucleotides (LNA-YN8-A and LNA-YN8-B) were modified with locked nucleotide acids. The gene amplified using the primer pair ACTB-F and ACTB-R is beta-actin from *Macaca mulatta* (African green monkey). The NCBI Reference Sequence for mRNA of beta-actin is NM_001033084.1. The gene amplified using the primer pair ORF7-F and ORF7-R is ORF7 from porcine reproductive and respiratory syndrome virus isolate YN-1 (NCBI Reference Sequence for the complete genome of YN-1 is GenBank: KJ747052.1).

**Table 1:** List of oligonucleotides used in this study

Name of oligonucleotides	Sequence (from 5′ to 3′)	Target gene	Position within the target gene	GC content (%)	Tm (°c)
YN8	TGCAGCATCCTCACAACCGT	Nsp9	704–723 bp	55	65.3
LNA-YN8-A	**TGCA**GCATCCTCACAA**CCGT**				71.8
LNA-YN8-B	**TG**CA**GC**ATC**CT**CAC**AA**CC**GT**				71.2
ACTB-F	agttgcgttacaccctttcttga	beta-actin	1168–1190	43.5	58
ACTB-R	tgctgtcaccttcaccgttc		1297–1316	55	59.4
ORF7-F	aatggccagccagtcaatca	ORF7	14,844–14,863	50	58.5
ORF7-R	tcacgctgagggcgatgctg		15,154–15,173	65	65

### Virus infection and transfection

Cell seeding, virus infection, transfection with AS-ONs and lifetime measurement of AS-ONs were performed [5]. In brief, the Marc-145 cells or PAMs were seeded in 96- or 6-well plates one day before PRRSV infection and AS-ON transfection. After inoculation with PRRSV YN1 strain (25 TCID_50_/well) for one and half hours, the medium was removed and transfection with the desired concentrations of AS-ONs was performed. Four hours post transfection, the transfection medium was replaced with fresh full DMEM medium till further analysis. Each treatment was performed in triplicate.

Cy-3 labeled AS-ONs were used in the transfection for the measurement of the lifetime of AS-ONs in vitro, with cy-3 fluorescence signals recorded under the fluorescence microscopy (Olympus) at various time points (1, 2, 6, 8 and 10 h post transfection).

### Cell viability analysis

The Marc-145 cells (10^4^ cells/well in 100 μl) were seeded into 96-well plates and incubated for overnight. Transfection with the indicated concentrations of AS-ONs (32, 40, 48, 56, 64 and 72 μM) was performed as described above, with each treatment in triplicate. Seventy-two hours post transfection, the cell viability analysis using CCK-8 kit (Sigma-Aldrich, Cat. No. 96992) was performed according to the manufacturer’s guide. In brief, 10 μl of CCK-8 solution was added to each well of the plate. Incubate the plate for 2 h in the incubator and measure the absorbance at 450 nm using a microplate reader. The wells without transfection were used as control for normalization.

### Isolation of total RNA, reverse transcription and qPCR analysis

As we previously described [5], total RNA was isolated from Marc-145 cells or PAMs approximately 60 h post PRRSV infection and AS-ON transfection using the RNAiso Plus RNA isolation kit (Takara Dalian, China), and subjected to reverse transcription (Takara PrimerScript RT reagents kit) and qPCR analysis (SYBR Primer Ex Taq II kit). β-actin served as an internal control. The primer pairs used in this study are listed in Table 1. The ΔΔCt method [27] for relative quantification of gene expression was applied to determine viral RNA levels using SYBR Green real-time PCR. The relative amount of PRRSV RNA was normalized to β-actin mRNA. Amplification and detection of samples were performed with the CFX96 Touch Real-Time PCR Detection System (Bio-Rad, USA).

### Western blot analysis

Total viral and cellular proteins were isolated from PAMs in 6-well plates approximately 60 h post PRRSV infection with or without AS-ON transfections. The cells were scraped after one time PBS wash and collected by centrifuge at 10,000 rpm for 5 min. Into the pellet 100 μl RIPA lysis and extraction buffer (ThermoFisher Scientific, Cat. no. 89900), 1 μl Halt Protease Inhibitor Cocktail (ThermoFisher Scientific, Cat. no. 78430) and 1 μl Halt Phosphatase Inhibitor Cocktail (ThermoFisher Scientific, Cat. no. 78420) was added. The pellet was lyzed by thorough pipetting followed by protein extraction at 4 °C for 15 min and then subjected to centrifuge at 10,000 rpm for 5 min. The protein-containing supernatants were collected, mixed with 20 μl loading buffer and 20 μl bromophenol blue (0.4%) and boiled for 10 min. The protein was stored at − 20 °C till further western blot analysis. Purified protein samples were resolved under reducing and denaturing conditions using sodium dodecyl sulfate-polyacrylamide gel electrophoresis (SDS-PAGE) and 8% Bis-Tris Novex NuPage gels in conjunction with running buffer. Resolved proteins were transferred to nitrocellulose membranes and blocked at room temperature for 1 h in PBST containing 5% (*w*/*v*) dehydrated milk and 0.05% Tween 20 with shaking. Viral N protein or cellular β-actin was probed by overnight incubation at 4 °C with rocking with the primary anti-N protein monoclonal antibody (VMRD, Cat. no. 080728–004, mouse origin) or with the primary anti- β-actin polyclonal antibody (Proteintech, Cat. No.20536–1-AP, rabbit origin). The antibodies were diluted in filtered 5% milk–PBST at a ratio of 1:500 (anti-N protein antibody) or 1:1,1000 (anti- β-actin). The following incubation at room temperature with racking was with secondary goat anti-mouse conjugated horseradish peroxidase (HRP) (Proteintech) or goat anti-rabbit-conjugated horseradish peroxidase (Proteintech) antibody (1:2000 dilution in filtered 5% milk–PBST). Subsequently, western blots were treated with chemiluminescent ECL Plus substrate (Pierce, Rockford, IL) and imaged using chemiluminescent film (Kodak, Rochester, New York).

### Indirect immunofluorescence staining

Sixty hours post PRRSV infection and transfection with AS-ONs, the Marc-145 cells were washed with PBS and fixed with 4% paraformaldehyde (PFA) at room temperature for 10–15 min. After three washes with PBS, the cells were permeabilized with PBS containing 0.3% Triton X-100 for 15 min and blocked with PBS containing 1% BSA for 2 h at 4 °C. The nuclei staining with 5 μg/ml of Hoechst 33,342 (Life Technology) was carried out for 20 min at room temperature. The cells were subsequently incubated at 4 °C overnight with 5 μg/ml of PRRSV antibody against N protein (encoded by ORF7) (VMRD, Cat. no. 080728–004, mouse origin), washed three times with PBS and incubated with Alexa Fluor 488 conjugated goat anti-mouse IgG (H + L) antibody (Proteintech, Cat. no. 861163) at 5 μg/ml for 1 h at 37 °C. After three times PBS wash, the cells were subjected to image analysis by fluorescence microscopy (Olympus). Images were processed to calculate the percentage of infected cells by ImageJ, which was downloaded from (https://imagej.nih.gov/ij/).

### Virus titration

Marc-145 cells were seeded into 96-well plates (10^4^ cells/well in 100 μl) one day before PRRSV infection and AS-ONs transfection. A 10× serial dilution of PRRSV YN-1 strain was prepared. Each dilution was added into six wells (100 μl/well). One and half hours post infection, the transfection was performed with 8 μM of AS-ONs. CPE was recorded using the inverted microscope over a period of 4 days post transfection. Cell number was counted and the 50% tissue culture infected dose (TCID_50_) was determined by Reed–Muench method.

### Statistical analysis

Statistical analysis was performed using GraphPad Prism 4.0 (GraphPad Software Inc., San Diego, CA, USA). Data were analyzed by using the t-test, with two-tailed distribution. *P* < 0.05 was considered statistically significant.

## Results

### LNA modification conferred longer lifetime to antisense oligonucleotides

**Fig. 1:**
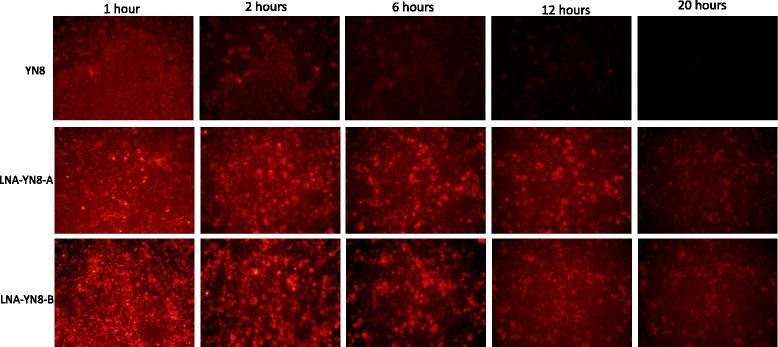
AS-ONs with LNA modifications were present in cell culture twenty hours post transfection. Cy3-labelled antisense oligonucleotides (YN8, LNA-YN8-A and LNA-YN8-B, 32 μM) were used in the transfection in Marc-145 cells. The fluorescent signals were recorded with fluorescence microscopy (Olympus) at the indicated time points post transfection. The fluorescent signals decreased over the time, however, compared with the DNA AS-ON YN8, we could clearly observe the signals even 20 h post transfection, indicating that incorporating LNA into the antisense oligonucleotide could improve the stability in Marc-145 cells for sufficiently longer time periods to induce the degradation of viral RNA and thus to inhibit PRRSV replication. Shown here are the representative images from three independent experiments

To investigate how long AS-ONs with LNA modifications are present in the cell culture system, 32 μM cy-3 labelled LNA modified antisense oligonucleotides LNA-YN8-A and LNA-YN8-B were transfected into Marc-145 cells. Transfection with cy-3 labelled DNA antisense oligonucleotide YN8 at the same concentration served as the control for comparison. At different time points (1, 2, 6, 12 and 20 h) post transfection, the medium was aspirated and the cells were washed with PBS prior to imaging in fresh medium (Fig. 1). We found that the fluorescent signal from cy-3 labelled antisense oligonucleotides containing LNA modification (LNA-YN8-A and LNA-YN8-B) was still detectable 20 h post transfection while the signal from cy-3 labelled DNA antisense oligonucleotide YN8 was not visible 6 h post transfection, indicating the unmodified oligonucleotide was almost completely degraded after 6 h. The data presented in Fig. 1 demonstrated that LNA modification can add significant biostability to antisense oligonucleotides in cell cultures or reduce their sensitivity to nucleolytic degradation in biological media, and that protection with LNA significantly stabilized the antisense oligonucleotides against nucleolytic attack, which is consistent with the previously reported data [28, 29].

### Antisense oligonucleotides containing LNAs showed lower cytotoxicity

**Fig. 2:**
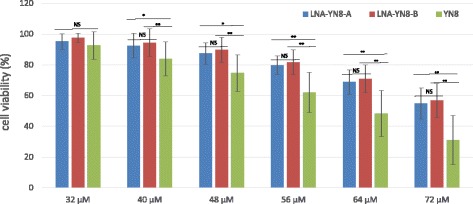
Cell viability analysis of Marc-145 cells transfected with AS-ONs containing LNA modifications. The cell viability was assayed 72 h post transfection. The measurement of each concentration was normalized to the corresponding cell control (as 100%). Compared with the cell control, both DNA AS-ON (YN8, when concentration ≥ 48 μM) and the AS-ONs with LNA modification (LNA-YN8-A and LNA-YN8-B, when concentration ≥ 56 μM) showed some cytotoxicity. Higher concentration led to higher cytotoxicity. However, LNA modification conferred lower cytotoxicity when compared with the corresponding DNA AS-ON YN8. No difference in cell viability was observed between LNA-YN8-A and LNA-YN8-B. Shown here are the representative data from three independent experiments. NS: not significant; *: *P* < 0.05; **: *P* < 0.01

The cell viability was assayed 72 h post transfection without infection using CCK-8 kit (Sigma-Aldrich, Cat. No. 96992) according to the manufacturer’s instructions. The cell viability of cell control with mock transfection but without PRRSV challenge was set as 100% for normalization and comparison. Compared with the cell control, both DNA AS-ON (YN8) and the AS-ONs with LNA modification (LNA-YN8-A and LNA-YN8-B) showed dose-dependent cytotoxicity. The data shown in Fig. 2 demonstrated that LNA modification conferred lower cytotoxicity when compared with the corresponding DNA AS-ON YN8. The cell viability from transfection with approximate 64 μM of LNA-YN8-A or LNA-YN8-B was similar with that from 48 μM YN8. No difference in cell viability was observed between the two LNA modified antisense sequences. The cell viability data implied that the antisense oligonucleotides with LNA modifications do not show obvious interference with cell viability with the concentration below 64 μM. Therefore the working concentrations of AS-ONs used in this study would not be toxic to the cells, excluding the possibility of inhibitory effects due to the toxicity of different AS-ONs.

### LNA modification protected Marc-145 cells from CPE induced by PRRSV infection

**Fig. 3:**
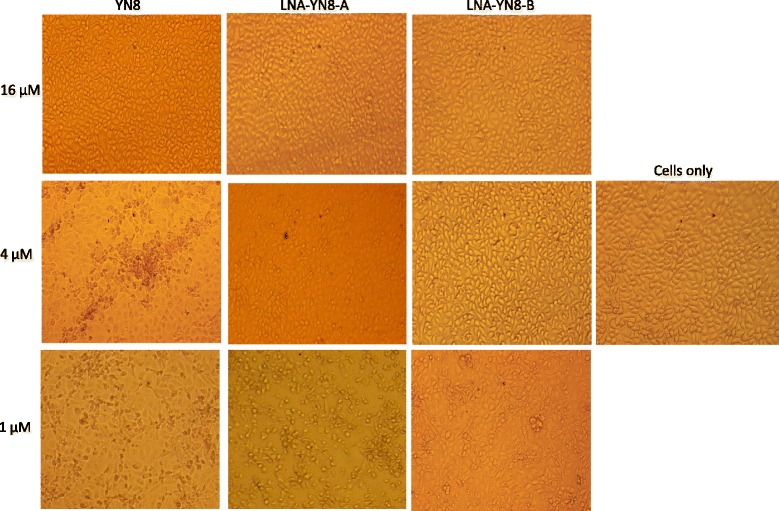
Cytopathic effect (CPE) analysis of Marc-145 cells transfected with AS-ONs containing LNA modifications. Compared with the mock treatment (bottom panel), transfection with 16 μM DNA antisense oligonucleotide YN8 or 4 μM LNA-YN8-A and LNA-YN8-B showed significant protection of Marc-145 cells from PRRSV infection, while transfection with lower concentrations (4 or 1 μM of YN8, 1 μM of LNA-YN8-A or LNA-YN8-B) did not. Compared with the normal cells (cells only, neither PRRSV inoculation nor transfection was applied), the cells in the YN8 wells (4 or 1 μM) and 1 μM LNA-YN8-A (or LNA-YN8-B) wells aggregated, rounded up and detached from the monolayer, while the cells transfected with YN8 (16 μM) or LNA modified YN8 (16 or 4 μM) manifested overtly less CPE. Pictures were taken 72 h post infection with a Nikon E5400 camera mounted on an inverted microscope (Nikon TS100). Shown here are the representative images from three independent experiments

To investigate whether AS-ONs with LNA modifications can further protect Marc-145 cells from the cytopathic effect (CPE) induced by PRRSV, one and half hours post challenge with 25 TCID_50_ PRRSV, Marc-145 cells were transfected with LNA-YN8-A and LNA-YN8-B at different concentrations (16 μM, 4 μM and 1 μM). Transfection with YN8 and cells free of PRRSV infection and transfection (cells only) served as the controls for comparison. The CPE image for each treatment shown in Fig. 3 was acquired 72 h post transfection.

In Fig. 3, no significant cytotoxic effects were observed in the mock well or in the transfection well with YN8 (16 μM), LNA-YN8-A or LNA-YN8-B (16 or 4 μM for both LNA modified AS-ONs). However, the CPE was manifested in the transfection wells with YN8 (4 or 1 μM), LNA-YN8-A or LNA-YN8-B (1 μM). The data here indicates that 16 μM of DNA AS-ONs is protective for Marc-145 cells from PRRSV infection, which brought the working concentration (32 μM) [5] down to half (16 μM), while the inhibitory dose for AS-ON with LNA modification (LNA-YN8-A and LNA-YN8-B) is 4 μM. The CPE data suggested that the antisense oligonucleotides with LNA modifications were more potent in inhibiting PRRSV replication in Marc-145 cells.

### Antisense oligonucleotides containing LNAs further inhibited PRRSV replication in PAMs

**Fig. 4:**
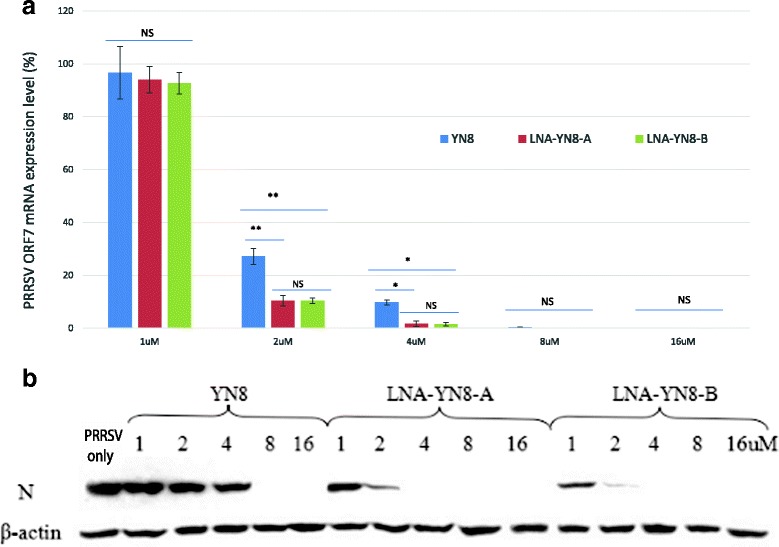
Viral ORF7 level was further reduced by transfection with LNA modified AS-ONs in PAMs. Antisense oligonucleotides (YN8, LNA-YN8-A and LNA-YN8-B) were used in the transfection in PAMs with different concentrations. The RNA (**a**) and protein (**b**) levels of gene ORF7 were more significantly inhibited by the treatments with LNA modified YN8 than by DNA AS-ON YN8 transfection. After virus challenge and transfection with AS-ONs, total RNA was isolated for RT-qPCR analysis and the total protein was extracted for western blot analysis. The ΔΔCt method for relative quantification of gene expression was used to determine viral ORF7 RNA levels. The Y-axis (**a**) shows the relative RNA levels of the ORF7 gene for each treatment after normalization to the non-transfected reference sample (PRRSV only). Transfection with 8 μM YN8 or 4 μM LNA-YN8-A or LNA-YN8-B completely blocked the synthesis of viral protein (**b**).The histogram and blots shown here are representative data from three independent experiments. β-actin was used as internal control in both RT-qPCR and western blot analysis. NS: not significant; *: P < 0.05; **: P < 0.01

To investigate whether AS-ONs with LNA modification could further inhibit PRRSV replication in porcine alveolar macrophages (PAMs), which are the targets of PRRSV in the porcine lung, PAMs were infected with PRRSV in vitro and 90 min later transfected by the two AS-ONs containing LNA modifications at different doses (1 μM, 2 μM, 4 μM, 8 μM and 16 μM). PAMs transfected with DNA AS-ON YN8 at the same concentrations and challenged with PRRSV (25 TCID_50_/well) were used as the control. Total RNA and protein was extracted from each treatment well 60 h post infection and subjected to RT-qPCR and western blot analysis, respectively. When normalized to β-actin for both mRNA and protein levels, the ORF7 RNA (Fig. 4a) and protein (Fig. 4b) levels in the PAMs transfected with any of the three AS-ONs were reduced in a dose dependent manner. Furthermore, Fig. 4 showed that transfection with YN8 at 8 μM or LNA-YN8-A or LNA-YN8-B at 4 μM completely abrogated the PRRSV replications in PAMs and that transfection with only half of the amount (4 μM) of AS-ON YN8 containing LNA modification could achieve the same protective effects as with 8 μM DNA AS-ON YN8. In addition, LNA-YN8-A and LNA-YN8-B at 2 μM still showed moderate inhibition effects on PRRSV replication.

### LNA modification conferred additional reduction of viral protein

**Fig. 5:**
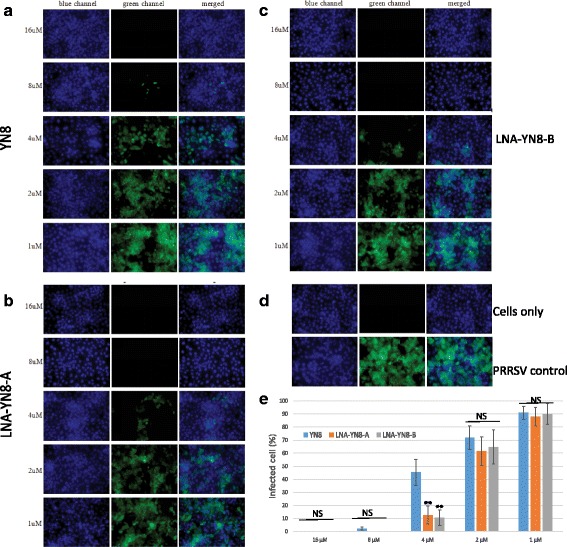
Indirect immunofluorescence detection of PRRSV in Marc-145 cells transfected with antisense oligonucleotides YN8 (**a**), LNA-YN8-A (**b**) or LNA-YN8-B (**c**). Cells with neither infection nor transfection and cells with infection but without transfection (**d**) serve as controls. Transfection was performed 90 min post infection with PRRSV YN-1 strain (25 TCID_50_). Cells were fixed 60 h post transfection. Anti-N monoclonal antibody (mAb) and Alexa Fluo488 conjugated secondary antibody were applied in the indirect immunofluorescence staining. Blue color stands for the nuclei and the green color indicates the expression of N protein in Marc-145 cells. The images of the same treatment from the two channels were merged. Treatment with LNA-YN8-A or LNA-YN8-B resulted in further reduction of virus replication, compared with the antisense oligonucleotide YN8 without LNA modifications. These images are representative for 3 independent experiments. Quantification data with statistical analysis of **a**, **b** and **c** was shown in **e**

To investigate the extra effect of AS-ONs containing LNA modification on inhibiting the expression of viral protein, indirect immunofluorescence assays were performed with anti-N protein mAb (encoded by gene ORF7) 60 h post transfection in Marc-145 cells with series dilution of AS-ONs (1, 2, 4, 8 and 16 μM). Indeed, as shown in Fig. 5, fewer fluorescing cells were seen in the monolayers treated with the two LNA modified AS-ONs than in the monolayers treated with the same amount of DNA AS-ON, and LNA-YN8-A or LNA-YN8-B at 4 μM displayed similar inhibition on viral N protein synthesis as the counterpart YN8 did at 8 μM, which is concordance with the RT-qPCR and western blot data from PAMs (Fig. 4).

### Reduction of viral titer by transfection with antisense oligonucleotides with LNA modifications

**Fig. 6:**
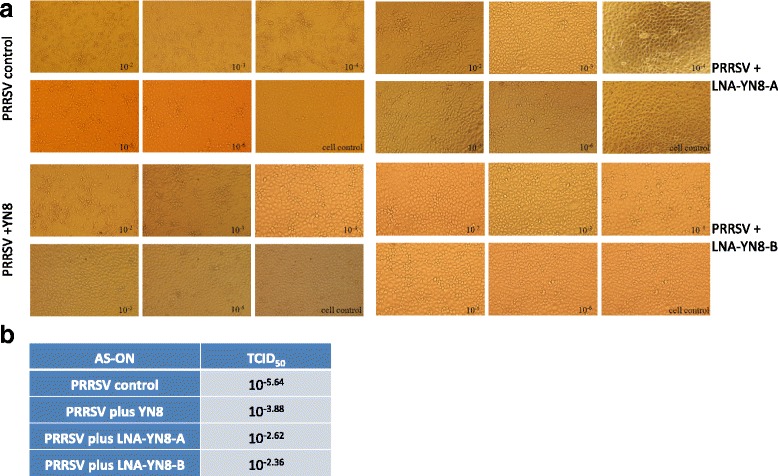
The antisense oligonucleotides with LNA modification could further protect Marc-145 cells from cytopathic effects and reduce the viral titer 10 fold, compared with the DNA AS-ON YN8. Marc-145 cells (10,000 cells per well) were seeded into 96-well plates the day before infection and transfection. Ten-fold serial dilution of PRRSV YN-1 strain was prepared with medium. 100 μl/well of each dilution was added into 6 wells in total. Ninety minutes post infection, the transfection was performed. CPE was recorded using the inverted microscope over a period of 4 days post transfection. The 50% tissue culture infected dose (TCID_50_) was determined by Reed–Muench method. Comparing to the DNA AS-ON YN8 control groups, transfection with LNA-YN8-A and LNA-YN8-B more significantly protected Marc-145 cells from cytopathic effects (**a**) and reduced the viral titer 10-fold (**b**). These data is representative for 3 independent experiments

Change of viral titer is one of the most direct and convincing parameters in anti-virus research. In order to further determine the level of inhibition, PRRSV YN-1 strain was diluted by 1:10 and added into Marc-145 cells. Transfection with 8 μM of the three AS-ONs was performed 90 min post infection, respectively. CPE was monitored until 4 days post virus infection and transfection. Viral titers were measured by TCID_50_ assay. We found that compared to the DNA AS-ON YN8 group, transfection with antisense oligonucleotide sequences containing LNA modifications (LNA-YN8-A or LNA-YN8-B) could more significantly protect Marc-145 cells from cytopathic effects (Fig. 6a) and reduced the viral titer by an extra 10-fold (Fig. 6b).

## Discussion and conclusion

Antisense therapy technology has been flourishing as a powerful therapeutic approach in the last 30 years with high expectations, exemplified by Merck’s acquisition of Sirna Therapeutics in 2006. More than 130 clinical trials are listed on https://clinicaltrials.gov/ (access data January 25th, 2017), with three approved antisense drugs (Vitravene, Kynamro and Macugen) and several others under consideration for market approval [30]. Among the efforts to improve the efficacy of antisense oligonucleotides, LNA modification holds one of the most promising technologies in antisense oligonucleotide therapeutics, as LNAs show higher thermostability and hybridization abilities, DNA nuclease resistance, activation of RNase H activity, solubility and penetration into the cells, lower cytotoxicity and easy synthesis [6, 7, 31]. Among the different designs of LNA modifications in antisense oligonucleotides, a number of studies revealed that mix-mers and gap-mers showed the best target degrading effects [16, 32, 33].

PRRSV is recognized as one of the most important viruses for the swine industry, mainly due to its persistence in pigs for quite a long time after initial infection. In our previous study [5], we performed a small-scale screening with nine DNA based AS-ONs as candidates and found YN8 to be the best sequence. To explore the advantages of LNA, in this study we incorporated LNA modifications into the YN8 sequence as gap-mer (LNA-YN8-A) and mix-mer (LNA-YN8-B) and tested the inhibitory effects of these two AS-ONs in cell cultures compared with the corresponding DNA based AS-ON YN8. The Marc-145 cells or PAMs were challenged with PRRSV YN-1 strain and transfection with the three AS-ONs, respectively, followed by lifetime analysis, cytotoxicity measurement, CPE observation, RT-qPCR, western blot analysis and TCID_50_ determination.

Taken together, under the experimental conditions in this study, we demonstrated that compared with the YN8 sequence, both gap-mer and mix-mer with LNA modifications showed longer lifetime (Fig. 1, 20 hs vs 6 hs), lower cytotoxicity (Fig. 2, 64 μM vs 48 μM) and higher protection from CPE induced by PRRSV infection (Fig. 3, 4 μM vs 16 μM), and more potent inhibitory effects on PRRSV replication in Marc-145 cells and PAMs at both mRNA and protein levels (2–4 μM vs 8 μM, Figs. 4, 5 and 6) with viral titer reduced by 10 fold. No significant difference was observed between the mix-mer and gap-mer. To our knowledge the only studies with modified antisense oligonucleotides in PRRSV research used phosphorodiamidate morpholono oligomers (PPMOs) [34–37]. The researchers tested the inhibition of some PPMOs on PRRSV replication in CRL11171 cells and found that the optimal working concentration was approximately 16 μM. In comparison, the LNA modified oligomers we designed and tested in both Marc-145 cells and PAMs inhibited the PRRSV replication at lower concentrations (2–4 μM), which demonstrated the advantages of incorporation of LNAs into AS-ONs to inhibit virus replication.

When it comes to the actual in vivo application of AS-ONs in the therapeutic control of PRRSV as shown in recent reports [37–39], we plan to integrate the methylation of cytosine, phosphorothioate modification in the backbone and the locked nucleic acid (LNA) modifications to redesign the YN8 sequence followed by testing the inhibition of PRRSV replication in piglets by gymnotic delivery of antisense oligonucleotides [40]. We believe this kind of systematical investigation will pave the way to further address the issue of PRRS control.

## Abbreviations

AS-ONs: Antisense oligonucleotides
CPE: Cytopathic effect
FBS: Fetal bovine serum
h: Hour
hpi: Hours post infection
LNA: Locked nuclei acid
NSP: Non-structural protein
ORF: Open reading frame
PAMs: Pulmonary alveolar macrophages
PRRS: Porcine reproductive and respiratory syndrome
PRRSV: Porcine reproductive and respiratory syndrome virus
qPCR: Quantitative PCR
TCID_50_: 50% Tissue Culture Infective Dose
UTR: Untranslated region

## References

[1] Pejsak Z, Markowska-Daniel I (1997). Losses due to porcine reproductive and respiratory syndrome in a large swine farm. Comp Immunol Microbiol Infect Dis.

[2] Meulenberg Janneke JM (2000). PRRSV, the virus. Vet Res.

[3] Dea S, Gagnon CA, Mardassi H, Pirzadeh B, Rogan D (2000). Current knowledge on the structural proteins of porcine reproductive and respiratory syndrome (PRRS) virus: comparison of the north American and European isolates. Arch Virol.

[4] Wang FX, Wen YJ, Yang BC, Liu Z, Shi XC, Leng X, Song N, Wu H, Chen LZ, Cheng SP (2012). Role of non-structural protein 2 in the regulation of the replication of the porcine reproductive and respiratory syndrome virus in MARC-145 cells: effect of gene silencing and over expression. Vet Microbiol.

[5] Zheng L, Li X, Zhu L, Li W, Bi J, Yang G, Yin G, Liu J (2015). Inhibition of porcine reproductive and respiratory syndrome virus replication in vitro using DNA-based short antisense oligonucleotides. BMC Vet Res.

[6] Koshkin AA, Rajwanshi VK, Wengel J (1998). Novel convenient syntheses of LNA [2.2.1]bicyclo nucleosides. Tetrahedron Lett.

[7] Wahlestedt C, Salmi P, Good L, Kela J, Johnsson T, Hökfelt T, Broberger C, Porreca F, Lai J, Ren K, Ossipov M, Koshkin A, Jakobsen N, Skouv J, Oerum H, Jacobsen MH, Wengel J (2000). Potent and nontoxic antisense oligonucleotides containing locked nucleic acids. Proc Natl Acad Sci U S A.

[8] Fesenko EE, Heydarov RN, Stepanova EV, Abramov ME, Chudinov AV, Zasedatelev AS, Mikhailovich VM (2013). Microarray with LNA-probes for genotyping of polymorphic variants of Gilbert's syndrome gene UGT1A1(TA)n. Clin Chem Lab Med.

[9] Karmakar S, Hrdlicka PJ (2013). DNA strands with alternating incorporations of LNA and 2'-O-(pyren-1-yl)methyluridine: SNP-discriminating RNA detection probes. Chem Sci.

[10] Liu JP, Guerasimova A, Schwartz R, Lange M, Lehrach H, Nyársik L, Janitz M (2006). LNA-modified Oligodeoxynucleotide hybridization with DNA microarrays printed on Nanoporous membrane slides. Comb Chem High Throughput Screen.

[11] Liu JP, Drungowski M, Nyársik L, Schwartz R, Lehrach H, Herwig R, Janitz M (2007). Oligonucleotide fingerprinting of arrayed genomic DNA sequences using LNA-modified hybridization probes. Comb Chem High Throughput Screen.

[12] Karkare S, Bhatnagar D (2006). Promising nucleic acid analogs and mimics: characteristic features and applications of PNA, LNA, and morpholino. Appl Microbiol Biotechnol.

[13] Kakiuchi-Kiyota S, Whiteley LO, Ryan AM, Mathialagan N (2016). Development of a method for profiling protein interactions with LNA-modified antisense oligonucleotides using protein microarrays. Nucleic Acid Ther..

[14] Latorra D, Campbell K, Wolter A, Hurley JM (2003). Enhanced allele-specific PCR discrimination in SNP genotyping using 3′ locked nucleic acid (LNA) primers. Hum Mutat.

[15] Vilas Boas D, Almeida C, Sillankorva S, Nicolau A, Azeredo J, Azevedo NF (2016). Discrimination of bacteriophage infected cells using locked nucleic acid fluorescent in situ hybridization (LNA-FISH). Biofouling.

[16] Jepsen JS, Sørensen MD, Wengel J (2004). Locked nucleic acid: a potent nucleic acid analog in therapeutics and biotechnology. Oligonucleotides.

[17] Letertre C, Perelle S, Dilasser F, Arar K, Fach P (2003). Evaluation of the performance of LNA and MGB probes in 5′-nuclease PCR assays. Mol Cell Probes.

[18] Palmano S, Mulholland V, Kenyon D, Saddler GS, Jeffries C (2015). Diagnosis of Phytoplasmas by real-time PCR using locked nucleic acid (LNA) probes. Methods Mol Biol.

[19] Subramanian N, Kanwar JR, Kanwar RK, Krishnakumar S (2015). Targeting Cancer cells using LNA-modified aptamer-siRNA chimeras. Nucleic Acid Ther.

[20] Nielsen BS, Møller T, Holmstrøm K (2014). Chromogen detection of microRNA in frozen clinical tissue samples using LNA™ probe technology. Methods Mol Biol.

[21] Guenther DC, Kumar P, Anderson BA, Hrdlicka PJ (2014). C5-amino acid functionalized LNA: positively poised for antisense applications. Chem Commun (Camb).

[22] Tian K, Yu X, Zhao T, Feng Y, Cao Z, Wang C, Hu Y, Chen X, Hu D, Tian X, Liu D, Zhang S, Deng X, Ding Y, Yang L, Zhang Y, Xiao H, Qiao M, Wang B, Hou L, Wang X, Yang X, Kang L, Sun M, Jin P, Wang S, Kitamura Y, Yan J, Gao GF. Emergence of fatal PRRSV variants: unparalleled outbreaks of atypical PRRS in China and molecular dissection of the unique hallmark. PLoS One 2007;13;2(6):e526. 10.1371/journal.pone.0000526 PMC1885284 17565379

[23] Zhou L, Yang HC (2010). Porcine reproductive and respiratory syndrome in China. Virus Res.

[24] Bo X, Lou S, Sun D, Shu W, Yang J, Wang S (2006). Selection of antisense oligonucleotides based on multiple predicted target mRNA structures. BMC Bioinformatics.

[25] Mathews DH (2006). RNA secondary structure analysis using RNAstructure. Curr Protoc Bioinformatics.

[26] Reuter JS, Mathews DH (2010). RNAstructure: software for RNA secondary structure prediction and analysis. BMC Bioinformatics.

[27] Pfaffl MW (2001). A new mathematical model for relative quantification in real time RT-PCR. Nucleic Acids Res.

[28] Elayadi AN, Braasch DA, Corey DR (2002). Implications of high-affinity hybridization by locked nucleic acid oligomers for inhibition of human telomerase. Biochemistry.

[29] Sazani P, Kole R (2003). Therapeutic potential of antisense oligonucleotides as modulators of alternative splicing. J Clin Invest.

[30] Aartsma-Rus A (2016). New momentum for the field of oligonucleotide therapeutics. Mol Ther.

[31] Kurreck J, Wyszko E, Gillen C, Erdmann VA (2002). Design of antisense oligonucleotides stabilized by locked nucleic acids. Nucleic Acids Res.

[32] Fluiter K, ten Asbroek AL, de Wissel MB, Jakobs ME, Wissenbach M, Olsson H, Olsen O, Oerum H, Baas F (2003). In vivo tumor growth inhibition and biodistribution studies of locked nucleic acid (LNA) antisense oligonucleotides. Nucleic Acids Res.

[33] Jepsen JS, Pfundheller HM, Lykkesfeldt AE (2004). Down-regulation of p21 (WAF1/CIP1) and estrogen receptor α in MCF-7 cells by antisense oligonucleotides containing locked nucleic acid (LNA). Oligonucleotides.

[34] Zhang YJ, Stein DA, Fan SM, Wang KY, Kroeker AD, Xj M, Iversen PL, Matson DO (2006). Suppression of porcine reproductive and respiratory syndrome virus replication by morpholino antisense oligomers. Vet Microbiol.

[35] Patel D, Opriessnig T, Stein DA, Halbur PG, Meng XJ, Iversen PL, Zhang YJ (2008). Peptide-conjugated morpholino oligomers inhibit porcine reproductive and respiratory syndrome virus replication. Antivir Res.

[36] Han X, Fan S, Patel D, Zhang YJ (2009). Enhanced inhibition of porcine reproductive and respiratory syndrome virus replication by combination of morpholino oligomers. Antivir Res.

[37] Opriessnig T, Patel D, Wang R, Halbur PG, Meng XJ, Stein DA, Zhang YJ (2011). Inhibition of porcine reproductive and respiratory syndrome virus infection in piglets by a peptide-conjugated morpholino oligomer. Antivir Res.

[38] Stein CA, Hansen JB, Lai J, Wu SJ, Voskresenskiy A, Høg A, Worm J, Hedtja M, Souleimanian N, Miller P, Soifer HS, Castanotto D, Benimetskaya L, Ørum H, Koch T (2010). Efficient gene silencing by delivery of locked nucleic acid antisense oligonucleotides, unassisted by transfection reagents. Nucleic Acids Res.

[39] Torres AG, Threlfall RN, Gait MJ (2011). Potent and sustained cellular inhibition of miR-122 by lysine-derivatized peptide nucleic acids (PNA) and phosphorothioate locked nucleic acid (LNA)/2'-O-methyl (OMe) mixmer anti-miRs in the absence of transfection agents. Artif DNA PNA XNA.

[40] Soifer HS, Koch T, Lai J, Hansen B, Hoeg A, Oerum H, Stein CA (2012). Silencing of gene expression by Gymnotic delivery of antisense oligonucleotides. Methods Mol Biol.

